# The MoS_2_ Nanotubes with Defect-Controlled Electric Properties

**DOI:** 10.1007/s11671-010-9765-0

**Published:** 2010-09-03

**Authors:** Maja Remskar, Ales Mrzel, Marko Virsek, Matjaz Godec, Matthias Krause, Andreas Kolitsch, Amol Singh, Alan Seabaugh

**Affiliations:** 1 Jozef Stefan Institute, Jamova 39, 1000 Ljubljana, Slovenia; 2 Institute of Metals and Technology, Lepi Pot 11, 1000 Ljubljana, Slovenia; 3 Forschungszentrum Dresden-Rossendorf, Institut für Ionenstrahlphysik und Materialforschung, D-01314 Dresden, Germany; 4 Department of Electrical Engineering, University of Notre Dame, Notre Dame, IN 46556, USA

**Keywords:** Inorganic Nanotubes, MoS2, Conductivity, Defects, Mo6S4I6

## Abstract

We describe a two-step synthesis of pure multiwall MoS_2_ nanotubes with a high degree of homogeneity in size. The Mo_6_S_4_I_6_ nanowires grown directly from elements under temperature gradient conditions in hedgehog-like assemblies were used as precursor material. Transformation in argon-H_2_S/H_2_ mixture leads to the MoS_2_ nanotubes still grouped in hedgehog-like morphology. The described method enables a large-scale production of MoS_2_ nanotubes and their size control. X-ray diffraction, optical absorption and Raman spectroscopy, scanning electron microscopy with wave dispersive analysis, and transmission electron microscopy were used to characterize the starting Mo_6_S_4_I_6_ nanowires and the MoS_2_ nanotubes. The unit cell parameters of the Mo_6_S_4_I_6_ phase are proposed. Blue shift in optical absorbance and metallic behavior of MoS_2_ nanotubes in two-probe measurement are explained by a high defect concentration.

## Introduction

One-dimensional nanostructures such as nanorods, nanobelts, nanowires and nanotubes have attracted intensive attention due to their unique applications in mesoscopic physics and nanoscale devices. In analogy to graphite, nanoparticles of many two-dimensional inorganic compounds are unstable against folding and can form closed cage structures which are referred to as inorganic fullerene-like particles and inorganic nanotubes[1]. Recent discovery of MoS_2_ nanopods called "mama"-tubes [2] with MoS_2_ fullerene-like particles in-situ grown in a confined geometry of MoS_2_ nanotubes and coaxial MoS_2_ nanotube hybrids [3] have opened a new way of synthesis of MoS_2_ nanotubes from Mo_x_S_y_I_z_ ternary compounds, which allows for the production of mass quantities of nanomaterials. Weak van der Waals interactions among MoS_2_ molecular layers enable low-strength shearing and several possible stackings [2,4].

Molybdenum disulfide nanostructures are receiving considerable attention because of their potential applications as heterogeneous catalysts for desulfurization processes [5], hydrogen evolution [6,7], and as materials for thermoelectric applications [8]. MoS_2_ microplatelets have been used as a solid lubricant or as an additive in oil or grease for more than 60 years. Cage-like nanostructures, e.g. cylindrical MoS_2_ nanotubes, represent a new generation of lubricants with extremely low friction resulting from the size, small enough to turn microvoids and nanovoids of the objects in mechanical contact into lubricant reservoirs, and by the curved geometry of the nanoparticles, which put them into constantly parallel orientation with the counterpart surfaces. The orientation relationship has been proposed to explain the ultra-low friction measured on thin films composed of MoS_2_ fullerene-like particles even at high humidity [9]. Lubrication is strongly related to electronic properties, more precisely to the filling of Mo $d_{z^{2}}$ molecular orbitals [10]. Control of nanotube dimensions and the density of defects that influence transport properties are of great importance in the construction of nanoscale electronic devices and multifunctional materials.

The significantly lower molecular mass of MoS_2_ in comparison with WS_2_ is an advantage for many applications, although MoS_2_ nanotubes are found to be more difficult to fabricate. Several different growth techniques are used for the synthesis of multiwall MoS_2_ inorganic nanotubes, like sulfurization of molybdenium oxides [11,12] and chlorides [13] in a stream of H_2_S gas, thermal decomposition of (NH_4_)_2_MoS_4 _[14] and by the template method [15], hydrothermal synthesis [16] and chemical transport reaction using iodine as a transport agent [17]. Currently, most of the described techniques are not suitable for large-scale production of pure multiwall MoS_2_ nanotubes, which would possess a relatively uniform size.

In the present paper, we report on a synthesis that can be scaled up for bulk production of pure multiwall MoS_2_ nanotubes of lengths up to several tens micrometers and diameters up to 100 nm using groups of Mo_6_S_4_I_6_ nanowires as precursor crystals. The structural data are combined with optical absorbance and Raman scattering. In addition, two-probe current–voltage measurements were performed on a single nanotube.

## Experimental

### Sample Preparation

The Mo_6_S_4_I_6_ nanowires were fabricated in evacuated (10^-4^ Pa) quartz ampoules directly from molybdenum and sulfur powder (Aldrich, 99.98 %), and iodine flakes (99.999%, Aldrich) in a molar ratio of 6: 3: 9. The iodine was used as the transport agent in the chemical transport reaction, which took place in a two-zone horizontal furnace for 48 h under a temperature gradient of 5.5 K/cm. A fraction (5–10 wt.%) of the total synthesized material was transported to the low-temperature zone (1010 K) of the ampoule and grew in the form of a hairy foil composed of Mo_6_S_x_I_y_ nanowires with some traces of MoI_2_ at the interface with the quartz, while the material remaining at the hot zone (1123 K) appeared as a dark-brown powder. The stoichiometry of this remaining powder in the form of Mo_6_S_4_I_6_ nanowires was determined by wave dispersive analysis using a scanning electron microscope, Jeol JSM 6500F. These nanowires were used as the precursor material for transformation into MoS_2_ nanotubes by annealing at 1073 K in the reactive gas composed of 98 vol% of Ar, 1 vol% of H_2_S, and 1 vol% of H_2_ for 1 h. In a typical experiment, around 600 mg of the starting material was sulfurized and transformed into MoS_2_ nanotubes. The total mass of the starting material during the transformation was decreased for 40% due to the complete removal of iodine and its substitution by the lighter sulfur. X-ray powder diffraction and X-ray energy dispersive analysis of the end product reveal the iodine-free MoS_2_ compound.

### Characterization

The Mo_6_S_4_I_6_ precursor crystals and MoS_2_ nanotubes have been studied by high-resolution 200 keV Jeol 2010 F field-emission transmission electron microscopes (HRTEM) and scanning electron microscope FE-SEM, Supra 35 VP, Carl Zeiss. X-ray diffraction (XRD), optical absorption, Raman spectroscopy, and wave dispersive analysis (WDS) were used to characterize the obtained materials. X-ray diffraction (XRD) was performed at room temperature with a D4 Endeavor diffractometer (Bruker AXS) using quartz monochromator Cu Kα1 radiation source (*λ* = 0.1541 nm) and Sol-X energy dispersive detector. Angular range 2 θ was chosen from 6° to 73° with a step size of 0.04° and a collection time of 4 s. The samples were rotated during measurements at 6 rpm. Raman spectra were recorded in a micro-Raman 180° backscattering configuration on a Labram HR spectrometer with a spectral resolution of 1.5 cm^-1^ determined by the width of 3 CCD-pixels. For excitation, a frequency-doubled Nd:YAG 532 nm laser operated with 100 μW power on the sample was used. Under these conditions, heating or degradation effects were excluded. Transport properties were measured using an Agilent 4155 semiconductor parameter analyzer using on-wafer probing of two-terminal test structures.

## Results and Discussion

### The Mo_6_S_4_I_6_ Nanowires

**Figure 1:**
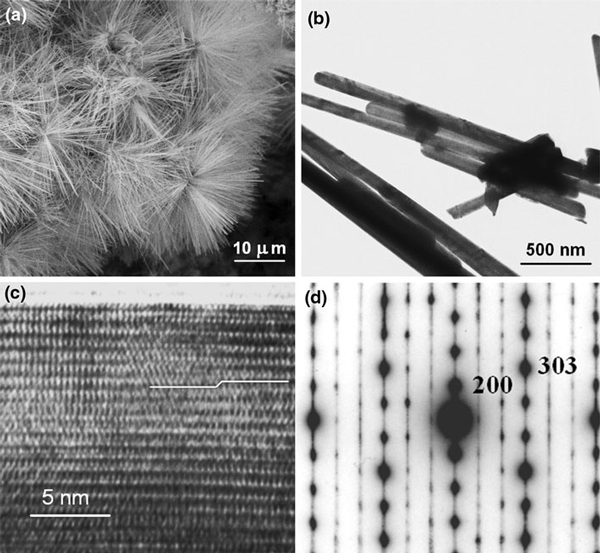
**The Mo_6_S_4_I_6_ nanowires: a A SEM image of hedgehog-like self-assemblies of identical nanowires grown up to 10 μm in length; b A TEM image revealing rigid nanocrystals with dome terminations; c A HRTEM image with *rows* of Mo_6_ clusters surrounded by sulfur and iodine atoms**. A stacking fault in otherwise regular order is marked with a stepped line and associated with a disordered structure; **d** TED pattern of a single Mo_6_S_4_I_6_ nanowire in the [010] zone assigned in accordance with the proposed space group P6_3_/m and lattice parameters of a hexagonal structure with: *a* = 1.88(5) nm and *c* = 1.18 nm.

Mo_6_S_4_I_6_ nanowires grew as hedgehog-like self-assemblies (Figure 1a) composed of nanowires of very homogeneous size, up to 100 nm in diameter and up to 20 μm in length. Considering that little information is available about this phase with no unit cell determined [18], we describe the direction of growth and assignment of the diffraction pattern in accordance with the similar Mo_6_S_2_I_8_ phase [19]. We find close similarities of electron and X-ray diffraction patterns of both phases, which generalize the report [20] on the stability of the Mo_6_S_9-*x*_I_*x*_ nanowires *(*4.5 <*x* < 6) with different S and I stoichiometries, to the Mo_6_S_4_I_6_ phase. Nanowires of different stoichiometries grow in skeletal structures composed of one-dimensional polymer chains of Mo_6_–chalcogen–halogen clusters, which differ only in the site occupation by sulfur and iodine. This makes difficulties in the determination of a particular phase, especially based on X-ray results. In our studies, we used electron diffraction obtained on a single nanowire for the elucidation of the symmetry rules, X-ray diffraction for the determination of interlayer distance with sufficient accuracy, and wave dispersive analysis for the determination of the stoichiometry on a single nanowire. Due to a mixed range of selective area diffraction, one cannot exclude the presence of other Mo_6_S_9-x_I_x_ and Mo_6_S_10-x_I_x_ nanowires in the starting materials, like Mo_6_S_3_I_6_ or Mo_6_S_2_I_8 _[20]. Nevertheless, most of the starting materials can be attributed to one phase, i.e. Mo_6_S_4_I_6_, while the others incorporate impurities that cause broadening of the X-ray peaks.

The Mo_6_S_4_I_6_ nanowires possess a high aspect ratio and grow in a longitudinal direction along the [001]. The needles are rigid and well crystallized (Figure 1b). One-dimensional chains are mutually ordered and in contrast with reported Mo_6_S_3_I_6_ nanowires do not exhibit a tendency for easy splitting. A stacking fault marked in Figure 1c with the component of the Burger's vector perpendicular to the nanowires axis can contribute to the resistance of the needles against longitudinal cleavage and decreases a strong anisotropy of these quasi one-dimensional cluster compounds.

The electron diffraction pattern of a single Mo_6_S_4_I_6_ nanowire (Figure 1d) is assigned in accordance with the proposed space group P6_3_/m and estimated lattice parameters of a hexagonal structure with: *a* = 1.88(5) nm and *c* = 1.18 nm. The nanowires grow with the [001] axis along their longitudinal direction.

**Figure 2:**
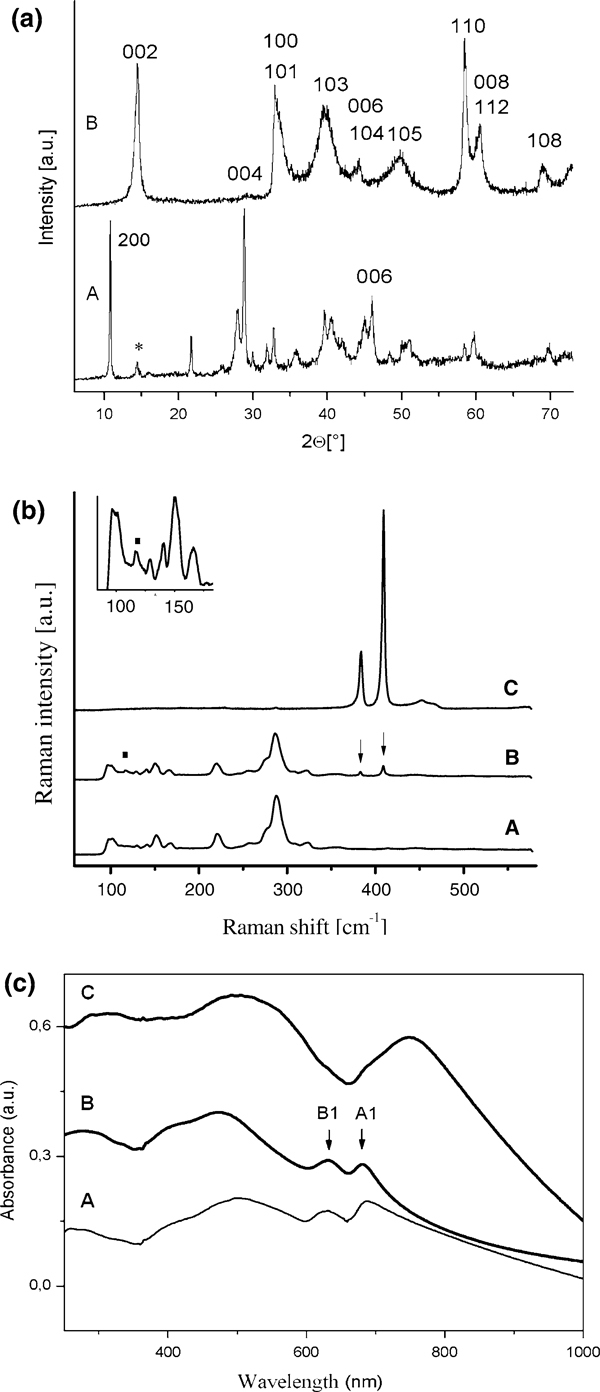
**a X-ray powder diffraction pattern (*A*) The starting material composed of Mo_6_S_4_I_6_ nanowires with traces of MoS_2_ (*asterisk*) and Mo_6_S_2_I_8_**. The two *indexed peaks* are used for approximation of the unit cell of Mo_6_S_4_I_6_; (*B*) The final material is composed of pure multiwall MoS_2_ nanotubes. **b** Raman spectra: (*A*) Mo_6_S_2_I_8_ nanowires, (*B*) Mo_6_S_4_I_6_ nanowires; *arrows* point traces of MoS_2_; square dot (see also the *inset*) shows a position of the characteristic peak at 117 cm^-1^, (*C*) MoS_2_ nanotubes gained by sulfurization of Mo_6_S_4_I_6_ nanowires. **c** Optical absorption spectra: (*A*) Mo_6_S_4_I_6_ precursor nanowires; (*B*) MoS_2_ nanotubes; (*C*) MoS_2_ polycrystalline material (*powder*). All materials were dispersed in ethanol.

Besides Mo_6_S_4_I_6_ nanowires, X-ray investigation of the starting material (Figure 2a-A) reveals the presence of the Mo_6_S_2_I_8_ and traces of MoS_2_. The (002) MoS_2_ peak is shown by an asterisk in spectrum (a), while other MoS_2_ peaks cannot be resolved. Due to nearly identical skeletal structures, most of the diffraction peaks of Mo_6_S_4_I_6_ and Mo_6_S_2_I_8_ nearly match, leading to a broadening of the peaks in addition to the size effect broadening. As an example, the peak at ~.817 nm is composed of two peaks situated at 0.816(1) nm and at 0.825(8) nm. The last one can be associated with the Mo_6_S_2_I_8_ (110) planes, while the first one was used for the approximation of the unknown unit cell of Mo_6_S_4_I_6_ nanowires. The symmetry of the TED pattern (Figure 1d) was utilized for assignation of the peak to the (200) planes of the Mo_6_S_4_I_6_. The peak positioned at 0.197 nm was associated with the (006) planes. Complete determination of the Mo_6_S_4_I_6_ structure needs further studies.

### Transformation of the Mo_6_S_4_I_6_ Nanowires into MoS_2_ Nanotubes

The X-ray powder diffraction of the product after the transformation (Figure 2a-B) reveals the iodine-free MoS_2_ compound. The spectrum is assigned according to the MoS_2_ (JCPDS-No. 77-1716). The Raman spectra of Mo_6_S_4_I_6_ nanowires are shown in Figure 2b-B and is almost identical with that of the pure Mo_6_S_2_I_8_ (Figure 2b-A). The only exception is the relative intensity of the peak at 117 cm^-1^, which is higher in the Mo_6_S_4_I_6_ compared to the Mo_6_S_2_I_8_. Some traces of MoS_2_ could also be observed in some spectra, marked by arrows. The spectroscopic similarity is a further support for the nearly identical crystal and local structure of Mo_6_S_4_I_6_ and Mo_6_S_2_I_8_. The Raman spectrum of the final product-MoS_2_ nanotubes (Figure 2b-C) contains the usual signature of MoS_2_, A_1g_ mode at 409 cm^-1^ and E_2g_ mode at 384 cm^-1 ^[21]. No traces of precursor Mo_6_S_4_I_6_ nanowires are observed. A small peak at the 287 cm^-1^ is attributed to the MoS_2_, E_1g_ mode that is forbidden in the backscattering experiments on the basal (001) plane. The reason for E_1g_ occurrence is the cylindrical geometry with a great part of the basal planes oriented in parallel with the axis of illumination. The line widths of the MoS_2_ nanotubes are larger than that of MoS_2_ single crystals. The FWHM of the A_1g_ line increases from 2.0 to 3.3 cm^-1^ and that of the E_2g_ from 1.6 to 3.8 cm^-1^. The line broadening is attributed to a smaller crystallite size and to a larger amount of defects in multiwall MoS_2_ nanotubes compared to MoS_2_ single crystals [22,23].

The electronic band structure calculations show that the bulk MoS_2_ is an indirect gap semiconductor with two exciton absorption bands at the absorption edge [24]. The absorption associated with the direct band gap is located in the visible spectrum around 700 nm and results from a direct transition at the K point. Two peaks assigned as A1 (690 nm) and B1 (620 nm) are attributed to two excitons of the Rydberg series. The band at around 500 nm is associated with the direct transitions from the valence band to the conduction band [25].

UV-Vis absorption spectroscopy was performed at room temperature. The MoS_2_ powder, Mo_6_S_4_I_6_ nanowires, and MoS_2_ nanotubes were ultra-sonicated in ethanol. MoS_2_ powder (Aldrich) was used as a reference material. The UV-Vis absorption spectrum (Figure 2c) of the Mo_6_S_4_I_6_ nanowires (A) reveals two broad peaks at 748 nm (1.66 eV) and 487 nm (2.55 eV). The absorption spectrum of the MoS_2_ nanotubes (B) reveals the A1 peak occurring at 681 nm and B1 at 631 nm with energy separation of 0.14 eV. The third peak dominates the spectrum, and it is blue-shifted with respect to MoS_2_. It is composed of a peak at 472 nm (2.63 eV) and its shoulder at 416 nm (2.99 eV). Comparison with the spectrum obtained by dispersed MoS_2_ powder in the form of platelets (C) reveals two main differences in the MoS_2_ nanotube sample: (i) a decrease in energy separation between A1 and B1 associated with the spin-orbit splitting of the top of the valence gap at the K point [20] and (ii) a blue shift, relative intensity and the shape of the absorption peak centered at the 472 nm, revealing changes in the direct transitions from the valence band to the conduction band. Besides these differences, the spectrum of MoS_2_ nanotubes matches well with the spectrum belonging to the dispersed MoS_2_ polycrystalline sample.

### Morphology of MoS_2_ Nanotubes

**Figure 3:**
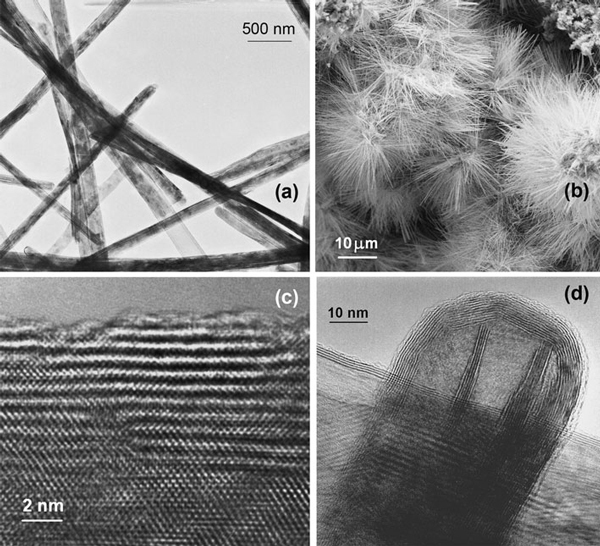
**The MoS_2_ nanotubes: a A typical TEM image of a bunch of nanotubes grown from sacrificed Mo_6_S_4_I_6_ nanowires keeping their outer geometry; and b self-organization in hedgehock-like groups revealed by SEM; c HRTEM image of a nanotube's wall with resolved MoS_2_ molecular layers, stacking faults and atomically rough surface; d TEM image of a single nanotube cap of a nanotube with inner cylinders terminated by curved layers of outer cylinders**.

The MoS_2_ multiwall nanotubes gained by the sulfurization after complete iodine removal keep the shape of the precursor Mo_6_S_4_I_6_ nanowires (Figure 3a). The nanotubes are still organized in hedgehog-like groups (Figure 3b). The tube walls with a typical thickness below 10 nm are strongly defected (Figure 3c). The lattice defects condensate forming faceting of the dome closure or disorder areas near stacking faults of MoS_2_ layers. Some inner cylinders can be terminated by curved players of outer cylinders (Figure 3d) revealing a dominant tendency of self-termination of surface molecular layers, while the inner ones did not gain sufficient energy for a closure.

### Transport Measurement on a Single MoS_2_ Nanotube

**Figure 4:**
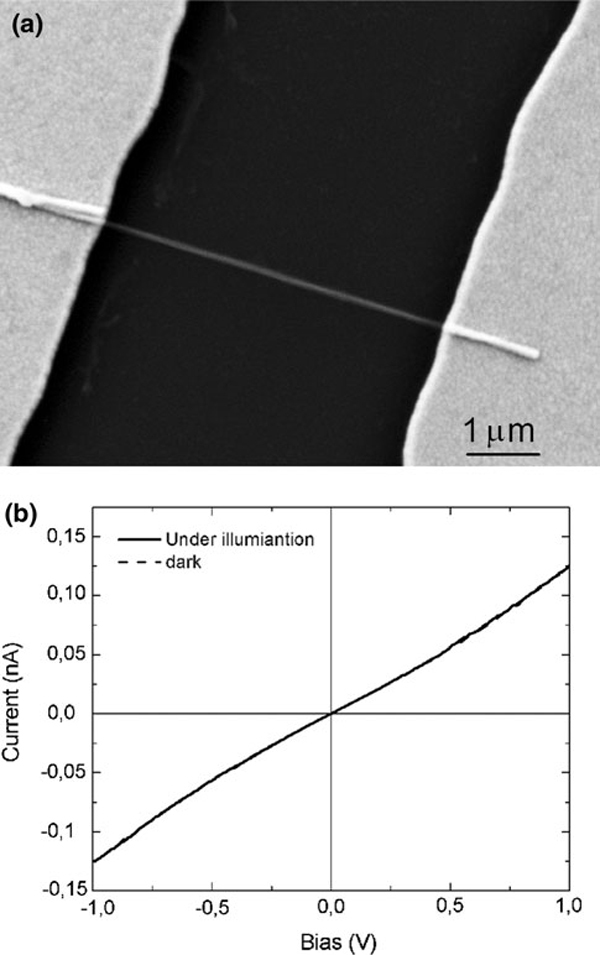
**a Scanning electron micrograph of a MoS_2_ nanotube between Ti/Au metal electrodes; b Current–voltage characteristics in the dark and under illumination for the MoS_2_ nanotube at room temperature with and without visible light illumination**.

Two-terminal device was fabricated from MoS_2_ nanotubes to measure transport properties (Figure 4a). The tubes were placed on a thermally-oxidized, heavily-doped p-Si substrate with a 90-nm-thick SiO_2_ overlayer. Titanium/gold contacts 20/220 nm in respective thickness were formed by electron beam evaporation using a photolithographic lift-off process with separations between the electrodes of 5 μm. Two-terminal measurements on a MoS_2_ nanotube, 105 nm in diameter, show a metallic (approximately ohmic) conduction. No photoconductivity was observed between measurements taken in the dark and under strong microscope illumination using a halogen lamp (Figure 4b). In addition, the wire conductivity could not be modulated independently on light illumination when backgated through the p + Si substrate in the range from -50 to +50 V with a tube bias ranging from 0 to 5 V, consistent with the metallic character of the nanotube. The nanotube conductivity is estimated to about 2 mS/cm by assuming that the wall thickness is 10 nm, which is the most frequently observed value in these nanotubes. The metallic conductivity could indicate a high density of defects, which form energy states near the Fermi level and influence also the optical absorbance spectra.

## Conclusion

In conclusion, we have reported an easy and straightforward way of fabricating pure multiwall MoS_2_ nanotubes using Mo_6_S_4_I_6_ nanowires as the precursor crystals. We synthesized the nanowires from elements in a furnace gradient and proposed the unit cell of the Mo_6_S_4_I_6_ compound, which was first reported in 1985. The nanotubes produced by the sulfurization process keep the outer geometry and self-assembly of the precursor nanowires. They are of relatively homogeneous size in diameter and length. The lattice structure is strongly defected causing Raman line broadening, a blue shift in visible light absorption and metallic conductivity at room temperature of this otherwise semiconducting compound. The synthesis, which can be easily scaled up, and peculiar energy level distribution in these MoS_2_ nanotubes could find application in nanoelectronics and tribology.

## References

[B1] Tenne R Nat Nanotechnol 2006 1 103 10.1038/nnano.2006.62 18654160 18654160

[B2] Remskar M Mrzel A Virsek M Jesih A Adv Mater 2007 19 4276 10.1002/adma.200701784

[B3] Remskar M Virsek M Mrzel A Apply Phys Lett 2009 95 133122 10.1063/1.3240892

[B4] Remskar M Skraba Z Ballif C Sanjines R Levy F Surf Sci 1999 433-435 637 10.1016/S0039-6028(99)00086-2

[B5] Lauritsen J Kibsgaard J Helveg S Topsoe H Clausen BS Lægsgaard E Besenbacher F Nat Nanotechnol 2007 2 53 10.1038/nnano.2006.171 18654208 18654208

[B6] Hinnemann B Moses PG Bonde J Jorgensen KP Nielsen JH Horch S Chorkendorff I Norskov JK J Am Chem Soc 2005 127 5308 10.1021/ja0504690 15826154 15826154

[B7] Jaramillo TF Jørgensen KP Bonde J Nielsen JH Horch S Chorkendorff I Science 2007 317 100 10.1126/science.1141483 17615351 17615351

[B8] Chiritescu C Cahill DG Nguyen N Johnson D Bodapati A Keblinski P Zschack P Science 2007 315 351 10.1126/science.1136494 17170252 17170252

[B9] Chhowalla M Amaratunga GAJ Nature 2000 407 164 10.1038/35025020 11001049 11001049

[B10] El Beqqali O Zorkani I Rogemond F Chermette H Ben Chaabane R Gamoudi M Guiilaud Synth Metals 1997 90 165 10.1016/S0379-6779(98)80002-7

[B11] Feldman Y Wasserman E Srolovitz DJ Tenne R Science 1995 267 222 10.1126/science.267.5195.222 17791343 17791343

[B12] Therese HA Zink N Kolb U Tremel W Solid State Sci 2006 8 1133 10.1016/j.solidstatesciences.2006.05.011

[B13] Deepak FL Margolin A Wiesel I Bar-Sadan M Popovitz-Biro R Tenne R Nano 2006 1 167 10.1142/S1793292006000173 21817676

[B14] Chen J Li SL Xu Q Tanaka K Chem Commun 2002 16 1722 10.1039/b205109e 12196967

[B15] Rivera-Munoz EM J Appl Phys 2007 102 094302 10.1063/1.2802292

[B16] Lavayen V Mirabal N O'Dwyer C Ana MAS Benavente E Torres CMS Gonzalez G Appl Surf Sci 2007 253 5185 10.1016/j.apsusc.2006.12.019

[B17] Remskar M Skraba Z Cleton F Sanjines R Levy F Appl Phys Lett 1996 69 351 10.1063/1.118057

[B18] Drobot DV Starkov VV Pisarev EA Russ J Inorg Chem 1985 30 1668

[B19] Perrin C Sergent M J Chem Res (S) 1983 38

[B20] Meden A Kodre A Padeznik Gomilsek J Arcon I Vilfan I Vrbanic D Mrzel A Mihailovic D Nanotechnology 2005 16 1578 10.1088/0957-4484/16/9/029

[B21] Sandoval SJ Yang D Frindt RF Irwin JC Phys Rev B 1991 44 8 10.1103/physrevb.44.3955 10000027

[B22] Virsek M Krause M Kolitsch A Mrzel A Iskra I Skapin SD Remskar M J Phys Chem C 2010 114 6458 10.1021/jp101298g

[B23] Frey GL Tenne R Matthews MJ Dresselhaus MS Dresselhaus G Phys Rev B 1999 60 4 2883 10.1103/PhysRevB.60.2883

[B24] Coehoorn R Hass C Dijkstra J Flipse CJF Phys Rev B 1987 35 6195 10.1103/PhysRevB.35.6195 9940850

[B25] Wilcoxon JP Newcomer PP Samara GA J Appl Phys 1997 81 7934 10.1063/1.365367

